# Carvacrol and *trans*-Cinnamaldehyde Reduce *Clostridium difficile* Toxin Production and Cytotoxicity *in Vitro*

**DOI:** 10.3390/ijms15034415

**Published:** 2014-03-12

**Authors:** Shankumar Mooyottu, Anup Kollanoor-Johny, Genevieve Flock, Laurent Bouillaut, Abhinav Upadhyay, Abraham L. Sonenshein, Kumar Venkitanarayanan

**Affiliations:** 1 Department of Animal Science, University of Connecticut, 3636 Horse Barn Hill Road Ext., Unit 4040, Storrs, CT 06269, USA; E-Mails: shaanvet@gmail.com (S.M.); kollanooranup@gmail.com (A.K.-J.); genevieve.flock@uconn.edu (G.F.); abhinav.upadhyay@uconn.edu (A.U.); 2 Department of Molecular Biology and Microbiology, Tufts University School of Medicine, Boston, MA 02111, USA; E-Mails: laurent.bouillaut@tufts.edu (L.B.); linc.sonenshein@tufts.edu (A.L.S.)

**Keywords:** plant compounds, *Clostridium difficile*, toxins, gene expression

## Abstract

*Clostridium difficile* is a nosocomial pathogen that causes a serious toxin-mediated enteric disease in humans. Reducing *C. difficile* toxin production could significantly minimize its pathogenicity and improve disease outcomes in humans. This study investigated the efficacy of two, food-grade, plant-derived compounds, namely *trans*-cinnamaldehyde (TC) and carvacrol (CR) in reducing *C. difficile* toxin production and cytotoxicity *in vitro*. Three hypervirulent *C. difficile* isolates were grown with or without the sub-inhibitory concentrations of TC or CR, and the culture supernatant and the bacterial pellet were collected for total toxin quantitation, Vero cell cytotoxicity assay and RT-qPCR analysis of toxin-encoding genes. The effect of CR and TC on a *codY* mutant and wild type *C. difficile* was also investigated. Carvacrol and TC substantially reduced *C. difficile* toxin production and cytotoxicity on Vero cells. The plant compounds also significantly down-regulated toxin production genes. Carvacrol and TC did not inhibit toxin production in the *codY* mutant of *C. difficile*, suggesting a potential *codY*-mediated anti-toxigenic mechanism of the plant compounds. The antitoxigenic concentrations of CR and TC did not inhibit the growth of beneficial gut bacteria. Our results suggest that CR and TC could potentially be used to control *C. difficile*, and warrant future studies *in vivo*.

## 1. Introduction

*Clostridium difficile* is a gram-positive, spore-forming, anaerobic bacterium that causes a toxin-mediated enteric disease in humans [1,2]. More than 300,000 cases of *C. difficile*-associated disease (CDAD) are reported annually in the United States, resulting in approximately US$3 billion of health care costs [3,4]. The emergence of a hypervirulent strain, NAP1/ribotype 027, that produces increased levels of toxins and a severe form of the disease in humans has been reported [5–7]. *C. difficile* predominantly affects long-term hospital inpatients and the elderly undergoing prolonged antibiotic therapy [6]. Prolonged antibiotic therapy results in the disruption of the normal enteric microflora, leading to the germination of *C. difficile* spores and pathogen colonization in the intestine with subsequent production of toxins [1,8]. The toxins, TcdA and TcdB, act as glucosyl transferases that inactivate the Rho family GTPases associated with F-actin regulation, and consequently cause disruption of the cytoskeleton and intestinal epithelial tight junctions [9,10]. This leads to an inflammatory response with the release of cytokines and leukotrienes, causing pseudomembrane formation and watery diarrhea [6,7,11]. The genes *tcdA* and *tcdB*, which encode the *C. difficile* toxins TcdA and TcdB, respectively, along with the genes encoding TcdR, an RNA polymerase sigma factor needed for maximal expression of *tcdA* and *tcdB*, TcdC, an antagonist of TcdR, and TcdE, a holin-like protein needed for toxin excretion, constitute the “pathogenicity locus” in *C. difficile* [11]. Expression of the pathogenicity locus is controlled by a number of environmental factors, including the availability of rapidly metabolizable carbon sources, a regulation mediated by the global regulator CcpA [12,13] and the intracellular pools of the branched-chain amino acids and GTP, mediated by the global regulator CodY [14,15]. Although exposure to broad-spectrum antibiotics predisposes patients to CDAD by disrupting the normal gut flora [16,17], antibiotics are still the primary line of treatment for patients who have contracted the disease. In addition, the emergence of antibiotic resistance in hypervirulent strains of *C. difficile* is increasingly reported worldwide [18,19]. Since *C. difficile* toxins are the major virulence factors responsible for the pathogenesis of CDAD, identification of alternative therapeutic agents that inhibit *C. difficile* toxin production without affecting the normal gastrointestinal flora or exacerbating bacterial antibiotic resistance could be a potentially viable approach for controlling CDAD.

Historically, plants have been used for treating various diseases in traditional medicine [20]. Carvacrol (CR) is a monoterpenoid phenol present in oregano and thyme oils. Diverse pharmacological actions of CR, including antimicrobial and anti-inflammatory properties, have been previously demonstrated [21]. *Trans*-cinnamaldehyde (TC) is an aromatic aldehyde present as a major component of the bark extract of cinnamon. Previous research conducted in our laboratory revealed that sub-inhibitory concentrations (SICs; the concentrations that do not inhibit bacterial growth) of TC and CR increased the sensitivity of multi-drug resistant *Salmonella* Typhimurium DT 104 to antibiotics by down-regulating antibiotic resistance genes and the efflux pump, *tolC* [20]. In addition, we previously observed that TC inhibited biofilm synthesis and virulence in uropathogenic *Escherichia coli* [22,23]. The objective of this study was to investigate the effect of sub-inhibitory concentrations (SICs) of TC and CR on toxin production and cytotoxicity of *C. difficile in vitro*, and delineate the potential mechanism(s) behind their effect.

## 2. Results and Discussion

### 2.1. Results

#### 2.1.1. Sub-Inhibitory Concentrations of CR and TC

**Figure 1.**
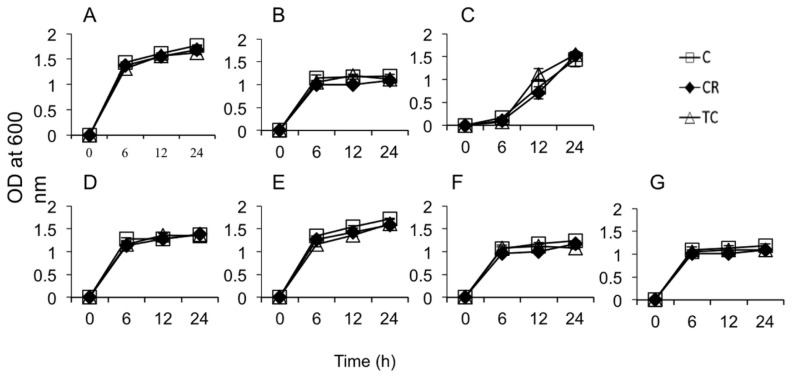
Effect of Sub-inhibitory concentrations of carvacrol (CR) and *trans*-cinnamaldehyde (TC) on beneficial gut bacterial growth. Seven selected beneficial gut bacteria (*Lactobacillus brevis* (**A**); *L. reuterii* (**B**); *L. delbrueckii bulgaricus* (**C**); *L fermentum* (**D**); *L. plantarum* (**E**); *Bifidobacterium bifidum* (**F**); and *Lactococcus lactis lactis* (**G**)) were grown in de Man, Rogosa and Sharpe broth in anaerobic condition at 37 °C with and without SICs of CR and TC (Control-Ctrl, open square, Carvacrol-CR, closed diamond, *trans*-cinnamaldehyde-TC, open triangle) for 24 h. The bacterial growth was monitored by measuring optical density at 600 nm measured at 6, 12 and 24 h. TC- or CR-treated gut bacterial populations did not change significantly from the controls (*p* > 0.05).

The SICs of CR and TC were found to be 0.60 mM (0.10 mg/mL) and 0.38 mM, (0.05 mg/mL), respectively. These concentrations of plant compounds did not inhibit the growth of all *C. difficile* isolates including CodY mutant and parental strains after 24 h incubation at 37 °C. In addition, the OD_600_ values for the seven selected beneficial bacterial isolates cultured in the presence of SICs of CR and TC were not significantly different from their respective controls (bacteria grown without CR or TC, Figure 1). This indicated that the concentrations of CR and TC used in this study were non-inhibitory to the growth of *C. difficile* (As shown in the Supplementary Figures S1–S3) as well as the seven selected beneficial bacteria.

#### 2.1.2. Effect of CR and TC on *C. difficile* Toxin Production

**Figure 2.**
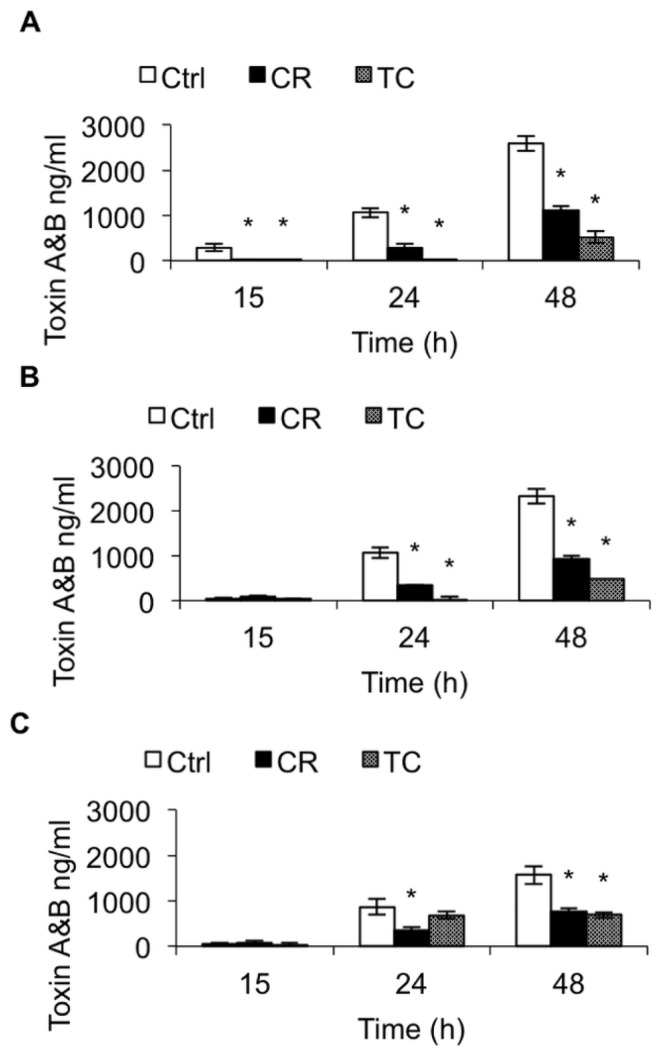
Effect of carvacrol (CR) and *trans*-cinnamaldehyde (TC) on *C. difficile* toxin production. * *p* < 0.05 *vs.* CR.

The effect of CR and TC on *C. difficile* toxin production was determined by ELISA, as described previously [24]. Carvacrol and TC significantly reduced toxin production in all three *C. difficile* isolates at 24 and 48 h (*p* < 0.05) compared to their controls. At 48 h, CR and TC inhibited the toxin production by approximately 60% and 80%, respectively in isolates BAA 1870 and 1053 (Figure 2A,B). In isolate BAA 1805, CR and TC reduced toxin production by approximately 55% at 48 h, compared to the control (Figure 2C). In isolates BAA 1870 and 1503, TC was more effective in reducing the toxin production than CR (*p* < 0.05) (Figure 2A,B), whereas both compounds exerted a similar inhibitory effect on toxin production in BAA 1805 after 48 h of incubation (Figure 2C).

Brain heart infusion with or without the Sub-inhibitory concentration (SIC) of TC or CR, (0.38 and 0.60 mM respectively) was inoculated (10^4^ CFU/mL) separately with three hypervirulent *C. difficile* isolates, ATCC BAA 1870 (A), ATCC BAA 1053 (B) or ATCC BAA 1805 (C), and incubated anaerobically at 37 °C for 72 h. The culture supernatant from groups TC, CR and Control (Ctrl) was collected at 15, 24, and 48 h of incubation for total toxin A and B quantitation by ELISA. * Treatments significantly differed from the control (*p* < 0.05).

#### 2.1.3. Effect of CR and TC on *C. difficile* Toxin-Mediated Cytotoxicity

**Figure 3.**
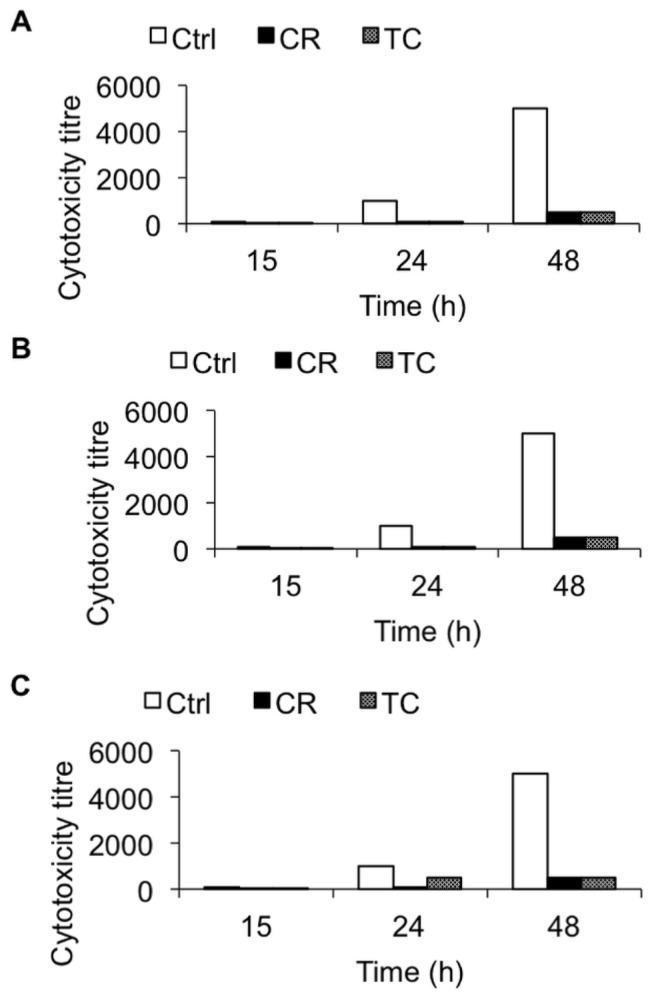
Effect of carvacrol (CR) and *trans*-cinnamaldehyde (TC) on *C. difficile* induced cytotoxicity on Vero cells.

To determine the efficacy of TC and CR in reducing *C. difficile*-induced cytopathic effects, a cytotoxicity assay was conducted on Vero cells following a previously published protocol [25]. Exposure to TC and CR significantly reduced the ability of *C. difficile* culture supernatant to produce cytotoxicity on Vero cells compared to culture supernatants of untreated cells (*p* < 0.05). The cytotoxicity titer in the cells inoculated with CR- and TC-treated *C. difficile* culture supernatant was reduced by ~90% at 48 h in comparison to that in the control (Figure 3).

Brain heart infusion with or without the Sub-inhibitory concentration (SIC) of TC or CR, (0.38 and 0.60 mM respectively) was inoculated (10^5^ CFU/mL) separately with three hypervirulent *C. difficile* isolates, ATCC BAA 1870 (A), ATCC BAA 1053 (B) or ATCC BAA 1805 (C), and incubated anaerobically at 37 °C for 72 h. The culture supernatant from groups TC, CR and Control (Ctrl) were collected at 15, 24, and 48 h of and the cytotoxicity titer on Vero cells was determined. Serially diluted *C. difficile* culture supernatants were added to the monolayers in 96-well plates and incubated at 37 °C under 5% CO_2_ for 48 h. The cell morphology was examined under an inverted microscope for characteristic rounding as an indication of cytotoxicity.

#### 2.1.4. Effect of CR and TC on *C. difficile* Toxin Genes

**Figure 4.**
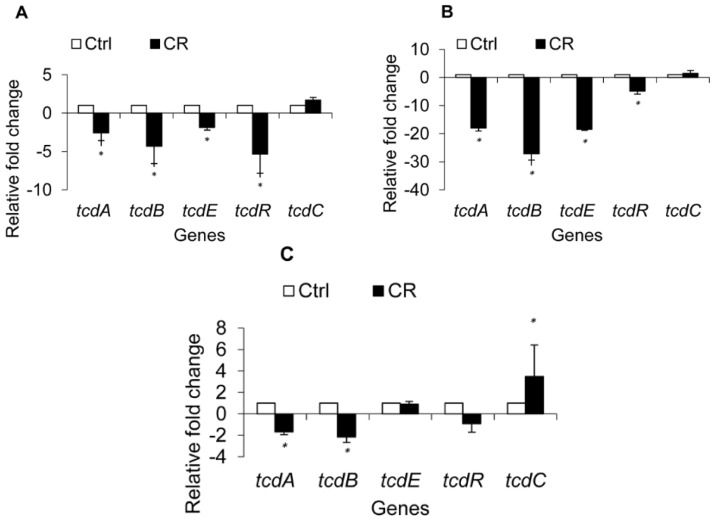
Effect of carvacrol (CR) on *C. difficile* toxin regulatory genes. Brain heart infusion with or without the sub-inhibitory concentration (SIC) of CR, (0.60 mM) was inoculated (10^5^ CFU/mL) separately with three hyper virulent *C. difficile* isolates, ATCC BAA 1870 (**A**); ATCC BAA 1053 (**B**) or ATCC BAA 1805 (**C**), and incubated anaerobically at 37 °C for 72 h. Bacterial pellets were harvested at 6 and 12 h for RNA isolation and RT-qPCR for toxin regulatory genes. * Treatments significantly differed from the controls (*p* < 0.05).

**Figure 5.**
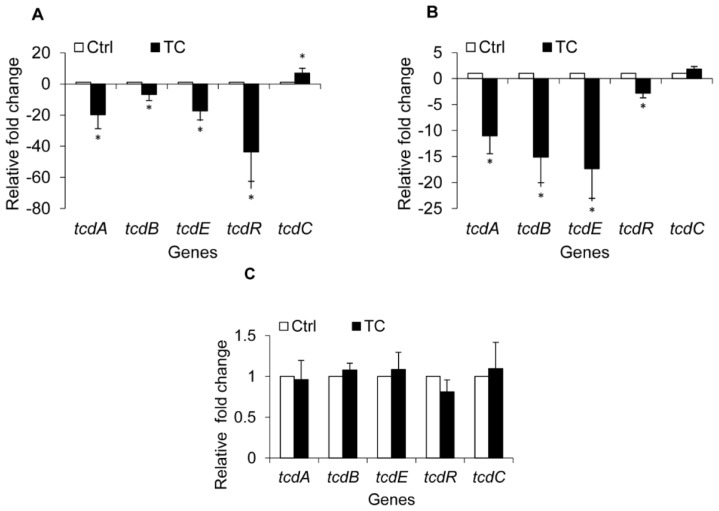
Effect of *trans*-cinnamaldehyde (TC) on *C. difficile* toxin regulatory genes. Brain heart infusion with or without the sub-inhibitory concentration (SIC) of TC, (0.38 mM) was inoculated (10^5^ CFU/mL) separately with three hyper virulent *C. difficile* isolates, ATCC BAA 1870 (**A**); ATCC BAA 1053 (**B**) or ATCC BAA 1805 (**C**), and incubated anaerobically at 37 °C for 72 h. Bacterial pellets were harvested at 6 and 12 h for RNA isolation and RT-qPCR for toxin regulatory genes. * Treatments significantly differed from the controls (*p* < 0.05).

To investigate if the observed reduction in *C. difficile* toxin production was due to modulation of the expression of toxin-associated genes, transcriptional analysis by RT-qPCR (Real time-quantitative polymerase chain reaction) of the genes known to be involved in toxin production and regulation was performed. The data revealed a modulation of *C. difficile* toxin regulatory genes by CR and TC at early stationary phase (12 h) (Figures 4 and 5). Carvacrol significantly down-regulated (*p* < 0.05) the expression of toxin genes *tcdA* and *tcdB* in all three isolates of *C. difficile* (*p* < 0.05) by ~2.5 fold (Figure 4A,C). However, in isolate BAA 1053, the genes were down-regulated by 15 to 25 fold (Figure 4B). Similar results were obtained in *C. difficile* isolates exposed to TC (Figure 5A,B). However, in isolate BAA 1805, no significant difference was observed in comparison to the control (Figure 5C). A significant down-regulation was also observed in the expression of the toxin excretion gene *tcdE* and the positive regulator *tcdR* in two *C. difficile* isolates (ATCC # BAA 1870, 1053) treated with CR and TC (Figures 4 and 5). In isolates BAA 1870 and 1805, *trans*-cinnamaldehyde treatment increased expression of *tcdC*, which encodes the TcdR antagonist (Figure 5A,B). Up-regulation of *tcdC* was observed in CR-treated BAA 1870 and 1053 as well (Figure 4A,B).

#### 2.1.5. Effect of CR and TC on a *codY* Mutant of *C. difficile*

**Figure 6.**
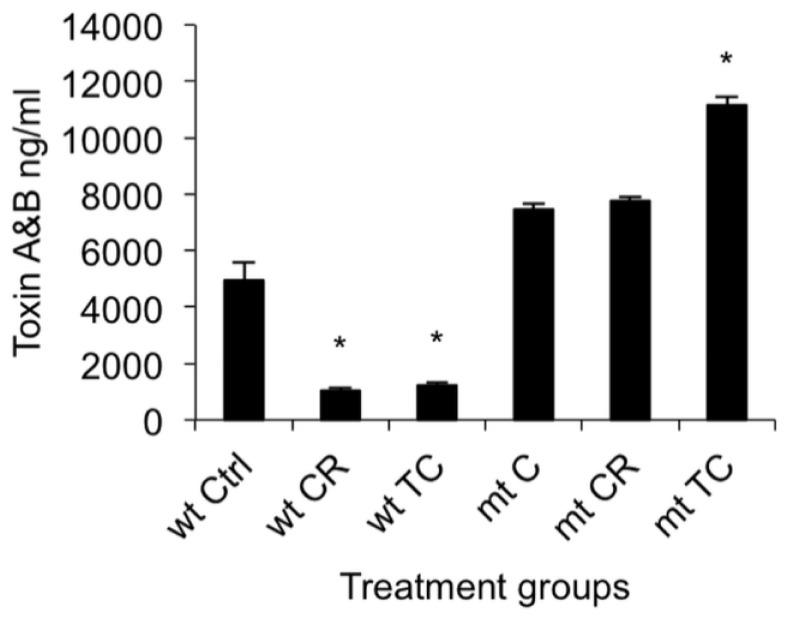
Effect of carvacrol (CR) and *trans*-cinnamaldehyde (TC) on *codY* mutant and wild type *C. difficile* toxin production. Brain heart infusion with or without the sub-inhibitory concentration (SIC) of TC or CR, (0.38 and 0.60 mM respectively) was inoculated (10^5^ CFU/mL) separately with UK1 and LB-CD16 (*codY* mutant) *C. difficile* isolates, and incubated anaerobically at 37 °C for 24 h. The culture supernatants from groups TC, CR and Control (Ctrl) were collected at 24 h of incubation for total toxin A and B quantitation by ELISA. * Treatments significantly differed from respective controls (*p* < 0.05).

In this experiment, we treated the *C. difficile codY* mutant (LB-CD16) and its parental strain, UK-1, with the SIC of CR or TC, and determined their effects on toxin production and toxin gene expression. The total toxin quantitation by ELISA (Enzyme Linked Immunosorbent Assay) revealed a significant reduction in toxin production in CR- or TC-treated UK-1 (*p* < 0.05) compared to the control (Figure 6). However, in a *codY* mutant, the level of toxin production in the absence of drug was higher than in the parent strain and neither CR nor TC caused any inhibition of toxin production. In fact, TC treatment enhanced the toxin production in the *codY* mutant strain significantly (*p* < 0.05) compared to the untreated control. Thus, in the absence of functional CodY protein, neither CR nor TC could inhibit toxin production.

**Figure 7.**
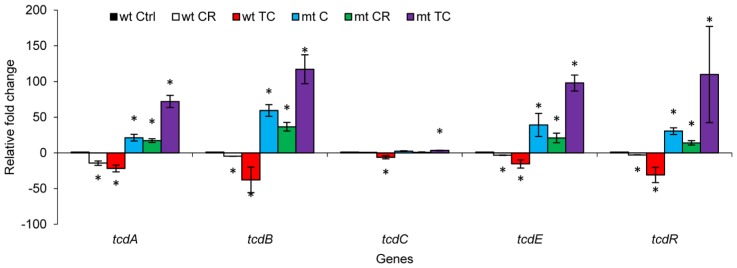
Effect of carvacrol (CR) and *trans*-cinnamaldehyde (TC) on *codY* mutant and wild type *C. difficile* toxin genes. Brain heart infusion with or without the sub-inhibitory concentration (SIC) of TC or CR, (0.38 and 0.60 mM respectively) was inoculated (10^5^ CFU/mL) separately with wild type (wt) UK-1 and its *codY* mutant (mt) *C. difficile* isolates, and incubated anaerobically at 37 °C for 72 h. Bacterial pellets were harvested at 6 and 12 h for RNA isolation and RT-qPCR for toxin regulatory genes. * Treatments significantly differed from respective controls (*p* < 0.05).

RT-qPCR analysis of the toxin genes in the *codY* mutant strain and its parent revealed a trend in gene expression similar to that seen for toxin production (Figure 7). A significant down-regulation of *tcdA* and *tcdB* was observed in strain UK-1 treated with CR or TC (*p* < 0.05). However, consistent with the ELISA results, CR and TC did not cause any down-regulation of the *tcdA* and *tcdB* genes in the *codY* mutant (*p* < 0.05). Specifically, no significant change in the expression of *tcdA*, *tcdB*, *tcdC* and *tcdR* was observed in the *codY* mutant treated with CR. However, TC treatment resulted in a significant up-regulation of *tcdA and tcdB* in the mutant (*p* < 0.05). These data collectively suggest that the SICs of CR and TC were unable to exert any inhibitory effect on the toxin-encoding genes in the absence of a functional *codY*.

### 2.2. Discussion

*C. difficile* colonizes the large intestine of patients undergoing prolonged antibiotic therapy, and produces toxins TcdA and TcdB, resulting in CDAD [1]. *C. difficile* toxins lead to inflammation in the intestine, increased epithelial permeability [26–28], enhanced cytokine and chemokine production [26,28], neutrophil infiltration [29] and the release of reactive oxygen intermediates [26], thereby causing direct damage to the intestinal mucosa [1]. Therefore, reducing toxin production by *C. difficile* is critical in controlling CDAD.

A potential alternate strategy for controlling microbial infections is the use of anti-virulence drugs. This class of drugs aims at reducing bacterial virulence rather than inhibiting bacterial growth [30] and presents a lesser selective pressure for development of bacterial antimicrobial resistance [31–33]. Therefore, in the current study, we investigated the efficacy of SICs of CR and TC as alternative therapeutic agents that can ameliorate CDAD pathology by reducing *C. difficile* toxin production without affecting the growth of the normal intestinal flora. Since the SICs of antimicrobials, including antibiotics, can modulate bacterial gene expression and physico-chemical functions, they have been used for studying effects on bacterial gene expression and virulence [34].

In the current study, we found that the SICs of CR and TC for *C. difficile* growth did not inhibit the growth of seven different species of bacteria commonly found in the human gastrointestinal tract. The tested gut bacteria included *Lactobacilli* and *Bifidobacteria*, which are the major groups of beneficial gut flora that play a significant role in maintaining normal gut health [35]. Previous studies have reported that CR and TC exerted no adverse effects on endogenous bacterial populations, including *Lactobacilli* and *Bifidobacteria*, in pigs and poultry [36,37].

Our ELISA results indicated that both plant compounds decreased the total toxin production in all three *C. difficile* isolates obtained from the ATCC, compared to their untreated control cultures (Figure 2). In addition, culture supernatants from TC- or CR-treated cells of all three strains had significantly reduced cytotoxic effects on Vero cells (Figure 3), confirming the ELISA results. Previously, Ultee and Smid (2001) reported that sub-MIC concentrations of CR decreased toxin production in *Bacillus cereus* [38]. Moreover, prior research conducted in our laboratory revealed that plant compounds, including CR and TC, reduce the virulence of *Listeria monocytogenes* by decreasing its attachment to and invasion of cultured human intestinal epithelial and brain micro-vascular endothelial cells by modulating the expression of several virulence factors, including the global regulator, *prfA* [39]. Since SICs of CR and TC were used in the experiments, the reduction observed in *C. difficile* toxin production in the treated samples was not due to growth inhibition of the bacterium by the plant compounds, but could be due to their potential abilities to modulate the expression of virulence genes associated with toxin production. Therefore, we used RT-qPCR to determine the effect of TC and CR on the transcription of major genes reported to play a role in toxin synthesis and secretion. Our RT-qPCR data revealed a 3-to-20-fold reduction in the expression of toxin-encoding genes *tcdA* and *tcdB* in strains BAA 1870 and BAA 1053. Furthermore, *tcdR*, which encodes a factor essential for high-level toxin production, was down-regulated 3-to-40-fold by CR and TC in those strains (Figures 4 and 5). Down-regulation of TcdR is known to lead to reduced toxin production [40]. Down-regulation of *tcdE* was also observed in these *C. difficile* isolates treated with CR or TC (Figures 4 and 5). Multiple studies have demonstrated that *tcdE* is critical for toxin release from *C. difficile*, helping to explain the reduced amount of toxins in CR- and TC-treated culture supernatants. In strain BAA 1805, however, the correlation between toxin and holin gene expression and toxin production was less clear suggesting that in this strain the modulation of toxin synthesis is mediated by a different mechanism than in other strains. Our results show that the compounds block toxin production in all strains, albeit to different extents, and that this effect can be attributed to decreases in toxin gene transcription in all strains except in the case of strain BAA 1805 in presence of TC.

CodY, a global regulatory protein that senses the intracellular levels of branched-chain amino acids and GTP, monitors the nutritional status and regulates metabolism and stress responses in Gram-positive bacteria [14,41,42]. In addition, CodY acts as a global regulator of virulence; a *codY* deletion results in enhanced virulence in *Staphylococcus aureus* and other species [42–45]. In *C. difficile*, CodY is a potent repressor of *tcdR*, thereby preventing expression of the Paloc genes when cells are growing rapidly [14,15]. Previous studies have shown increased expression of toxin genes in a *codY* mutant of *C. difficile* [14,15]. Our results show that CR and TC reduce toxin production and pathogencity locus gene expression in NAP1/027 strain UK-1, but not in a *codY* null mutant of UK-1. In fact, TC caused hyperexpression of toxin locus genes in the *codY* mutant strain. In a *codY* mutant strain, the compounds lose their effectiveness as inhibitors, suggesting that the compounds work by a mechanism that may affect either CodY synthesis or activity. CodY activity would be reduced if the compounds caused the pools of branched chain amino acids or GTP to drop [14,15]. These data collectively suggest that the antitoxigenic effect of CR and TC is mediated at least in part through CodY.

## 3. Experimental Section

### 3.1. Bacterial Strains and Culture Conditions

Four hypervirulent *C. difficile* isolates (ATCC# BAA 1870, 1053, 1805 and UK1) were grown in brain heart infusion broth (BHI) supplemented with 5% yeast extract (Difco, Sparks, MD, USA) in a Whitley A35 anaerobic work station (Microbiology Inc., Frederick, MD, USA) in the presence of 80% nitrogen, 10% carbon dioxide and 10% hydrogen at 37 °C for 24 h. The bacterial population was titered by plating 0.1 mL portions of appropriate dilutions on BHI agar and *Clostridium difficile* moxalactum norfloxacin (CDMN) agar (Oxoid, Hampshire, UK) supplemented with 5% horse blood, under strict anaerobic conditions at 37 °C for 24 h. In addition, seven selected beneficial enteric bacteria obtained from the USDA-ARS culture collection, Peoria, IL (*Lactobacillus brevis*, L*. reuterii*, L. *delbrueckii bulgaricus*, L. *fermentum*, L. *plantarum*, *Bifidobacterium bifidum*, and *Lactococcus lactis lactis*) were separately grown in de Man, Rogosa and Sharpe (MRS) broth (Difco, Sparks, MD, USA) under anaerobic conditions at 37 °C. The titers of the cultures were determined by plating 0.1 mL portions of appropriate dilutions on MRS agar (Difco, Sparks, MD, USA) with incubation at 37 °C for 24 h. The cultures were sedimented by centrifugation (3600× *g*, 15 min, 4 °C), and the pellets were washed twice, and resuspended in sterile phosphate-buffered saline (pH 7.3), and used as the inoculum.

### 3.2. Plant Compounds and SIC Determination

The SIC of TC and CR was determined as previously described [20]. Fifty ml of BHI supplemented with 5% yeast extract was inoculated separately with ~5.0 log_10_ CFU of each *C. difficile* isolate, followed by the addition of 1 to 10 μL of TC or CR (Sigma-Aldrich, St. Louis, MO, USA) with an increment of 1 μL. The cultures were incubated at 37 °C for 24 h, and bacterial growth was determined by measuring the optical density at 600 nm. The highest concentration of each plant compound that did not inhibit bacterial growth after 24 h of incubation was selected as its respective SIC (Sub Inhibitory Concentration) for this study.

Similarly, the effect of SIC of TC and CR on the growth of the aforementioned beneficial gut bacteria was determined by culturing them separately in 10 mL of MRS broth under anaerobic conditions at 37 °C with or without the plant compounds for 24 h. The growth of each culture was determined by measuring optical density at 600 nm, and plating on MRS agar.

### 3.3. Effect of Plant Compounds on *C. difficile* Toxin Production and Cytotoxicity

Brain Heart infusion broth, with or without the SIC of TC or CR was inoculated (10^5^ CFU/mL) separately with each *C. difficile* isolate, and incubated at 37 °C for 48 h anaerobically as before. The culture supernatant was collected at 15, 24, and 48 h of incubation for total toxin A and B quantitation by ELISA [24] and for determining cytotoxicity on Vero cells. The bacterial pellets were harvested at 6 and 12 h for RNA isolation and RT-qPCR analysis of *C. difficile* genes associated with toxin synthesis.

### 3.4. ELISA for Total Toxin A and B

The amount of toxin in the culture supernatant was quantified using the Wampole Tox A/B II kit (TechLabs, Inc., Blacksburg, VA, USA), as described by Merrigan *et al*. [24]. Purified toxin B (Sigma Aldrich, St. Louis, MO, USA) was used to plot a standard curve. The culture supernatants were diluted and ELISA was performed according to the manufacturer’s instructions. The optical density was measured at 450 nm, compared with the linear range of standard curve, and total toxin concentration was estimated.

### 3.5. Cytotoxicity Assay

The effect of CR and TC on the cytotoxicity of *C. difficile* culture supernatant was estimated by Vero cell cytotoxicity assay, as described previously [25]. *C. difficile* culture supernatant was serially diluted (1:10) and added onto Vero cell monolayers in 96-well microtiter plates. The plates were incubated at 37 °C in a 5% CO_2_ environment for 48 h, and examined under an inverted microscope. Positive reactions were indicated by the characteristic rounding of Vero cells accompanied by parallel neutralization of cytotoxicity with *Clostridium sordellii* antitoxin (TechLabs, Inc., Blacksburg, VA, USA). The cytotoxicity titer was expressed as the reciprocal of the highest dilution that caused more than 80% cell rounding.

### 3.6. Real Time Quantitative PCR (RT-qPCR)

In order to determine the effect of CR and TC on *C. difficile* genes involved in toxin synthesis, total RNA was isolated from the early stationary phase (12 h) cultures [5]. The culture supernatant was harvested by centrifugation at 3000× *g* for 10 min at 4 °C. The bacterial pellet was resuspended in RNAwiz solution (Ambion, Austin, TX, USA), flash frozen in liquid nitrogen, and stored at −80 °C. Total RNA extraction was performed using the Ambion RiboPure Bacteria RNA kit (Ambion, Austin, TX, USA) according to the manufacturer’s instructions, followed by DNase I digestion using Turbo DNase I (Ambion). The RNA obtained after each DNase I digestion was purified further using the Qiagen RNeasy RNA column purification kit, according to the manufacturer’s instructions (Qiagen, Germantown, MD, USA). The cDNA was synthesized using the Bio-Rad iScript cDNA synthesis kit (Bio-Rad, Hercules, CA, USA). RT-qPCR analysis of the genes associated with toxin production was performed using published primers for Paloc genes [46] normalized against 16S rRNA gene expression. Twenty-five μL reactions were performed in triplicate using iTaq SYBR (Bio-Rad, Hercules, CA, USA). The relative fold change in gene expression was calculated using the 2^−ΔΔ^*^C^*^t^ method [47].

### 3.7. Construction of a *codY* Mutant of *C. difficile* Strain UK-1 and the Effect of Plant Compounds on *codY* Mutant and Parental Strains

The pJS107 plasmid [48] was used as a TargeTron vector to introduce mutations into the NAP1/027 *C. difficile* strain UK-1. The group II intron insertion sites for *C. difficile codY* were identified using an algorithm that can be found at http://dna.med.monash.edu.au/~torsten/intron_site_finder/. The intron fragment was generated by PCR using primers oLB178 (TGAACGCAAGTTTCTA ATTTCGATTGTAGTTCGATAGAGGAAAGTGTCT), oLB179 (AAAAAAGCTTATAATTATCCT TAACTACCGTAGTAGTGCGCCCAGATAGGGTG), oLB180 (CAGATTGTACAAATGTGGTGA TAACAGATAAGTCGTAGTATATAACTTACCTTTCTTTGT) and EBS-Universal (CGAAATTA GAAACTTGCGTTCAGTAAAC), and a 1:1 mixture of pBL64 and pBL65 as template [49] and then cloned at the *Hind*III and *Bsr*GI sites of pJS107, yielding pBL103. pBL103 was then introduced into *B. subtilis* strain BS49 by transformation. Strain BS49 carries a conjugative transposon, Tn916, integrated into its chromosome and serves as a donor in conjugation with *C. difficile*. Conjugation experiments were carried out as described previously [50]. *C. difficile* transconjugants were selected on thiamphenicol (10 μg/mL) and then screened for the presence of Tn*916* by assaying tetracycline resistance (10 μg/mL). Thiamphenicol-resistant, tetracycline-sensitive (plasmid-containing, transposon-negative) transconjugants were selected for further use. Potential TargeTron mutants were identified by plating on lincomycin (20 μg/mL) and screening for the insertion of the intron into *C. difficile codY* by PCR using primers specific for full-length *C. difficile codY.* (oLB205, TGAGCATGCTTAATGATGATGATGATGATGTTGATTGTTTTTTAATTTTTTTAATTCATC; and oLB206, TATCCGGAATTCTGAGGAGATGATTAAATGGC), the 5′ intron insertion site and the 3′ intron insertion site and a positive clone, strain LB-CD16, was identified. A Southern blot was performed as previously described [49] to confirm that the mutant contained a single insertion of the intron.

The role of *codY* on the anti-toxigenic effect mediated by the plant compounds was investigated by quantifying toxin production and expression of toxin-associated genes in CR- and TC-treated *codY* mutant and wild type strains of *C. difficile*. Brain Heart infusion broth, with or without the SIC of TC or CR was separately inoculated (10^5^ CFU/mL) with UK1 or LB16, and incubated at 37 °C for 48 h, as described previously. The culture supernatants were collected at 24 and 48 h of incubation for total toxin A and B quantitation by ELISA. In addition, the bacterial pellets were harvested at 12 h for RNA isolation and RT-qPCR analysis.

### 3.8. Statistical Analysis

All experiments had duplicate samples and the study was repeated three times. The data were analysed using the PROC-MIXED procedure of SAS v. 9.3 (SAS Institute Inc., Cary, NC, USA). Differences between the means were considered significantly different at *p* < 0.05.

## 4. Conclusions

To conclude, our study demonstrated that CR and TC were effective in significantly reducing *C. difficile* toxin production. Although there were variations in the expression of toxin encoding genes, both plant molecules reduced toxin production in all strains (BAA 1870, BAA1053, BAA1805 and UK-1). The major toxin encoding genes, *toxA*, and *toxB* were significantly down-regulated in all the tested strains by CR, whereas TC down-regulated these genes in all strains except one (BAA 1805). Moreover, the two plant compounds did not inhibit the growth of major gut microflora in humans. The anti-toxigenic effect of CR and TC appears to be mediated through CodY. The results suggest the potential use of TC and CR to attenuate *C. difficile* virulence; *in vivo* studies on the effect of CR and TC on CDAD are thus warranted.

## Supplementary Information
