# Supplementary material for: Carvacrol and trans-Cinnamaldehyde Reduce Clostridium difficile Toxin Production and Cytotoxicity in Vitro
Source: Int J Mol Sci. 2014 Mar 12;15(3):4415–30. doi: 10.3390/ijms15034415 (PMC3975404; doi:10.3390/ijms15034415)
Supplement: Supplementary file 1 [file ijms-15-04415-s001.pdf]

Supplementary Information

Effect of Sub-inhibitory concentrations of carvacrol (CR) and trans-cinnamaldehyde (TC) *C. difficile* growth.

C. difficile isolates, ATCC BAA 1870 (S1), ATCC BAA 1053 (S2) or ATCC BAA 1805 (S3) were grown in brain heart infusion broth (BHI) supplemented with 5% yeast extract (Difco, Sparks, MD, USA) in a Whitley A35 anaerobic work station (Microbiology Inc., Frederick, MD, USA) in the presence of 80% nitrogen, 10% carbon dioxide and 10% hydrogen at 37 °C for 24 h with and without SICs of CR (0.60 mM) and TC (0.38 mM). The bacterial growth was monitored by serial dilution and plating in CDMN agar at 6, 12 and 24 h and expressed in colony forming units per mL. * The growth of TC (0.38 mM)—or CR (0.60 mM)-treated *C. difficile* did not change significantly from the controls ($p > 0.05$).

Figure S1. Effect of SIC concentrations of CR and TC on growth of C. difficile isolate ATCC BAA 1870. CFU/mL is Colony Forming Units/mL.

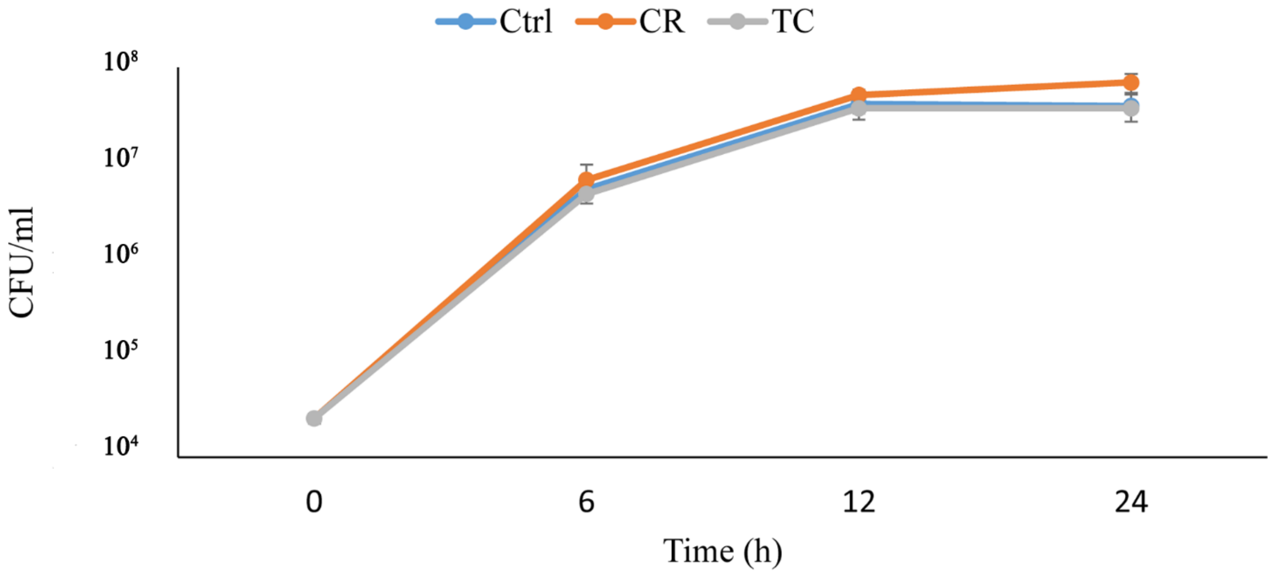

Figure S2. Effect of SIC concentrations of CR and TC on growth of *C. difficile* isolate ATCC BAA 1053.

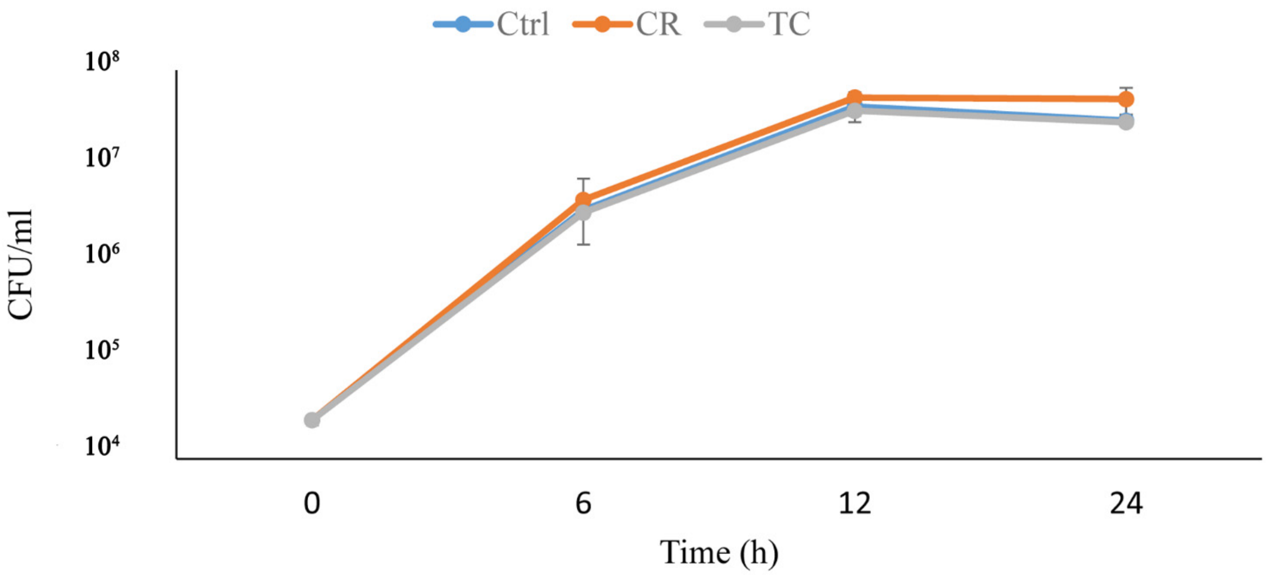

Figure S3. Effect of SIC concentrations of CR and TC on growth of *C. difficile* isolate ATCC BAA 1805.

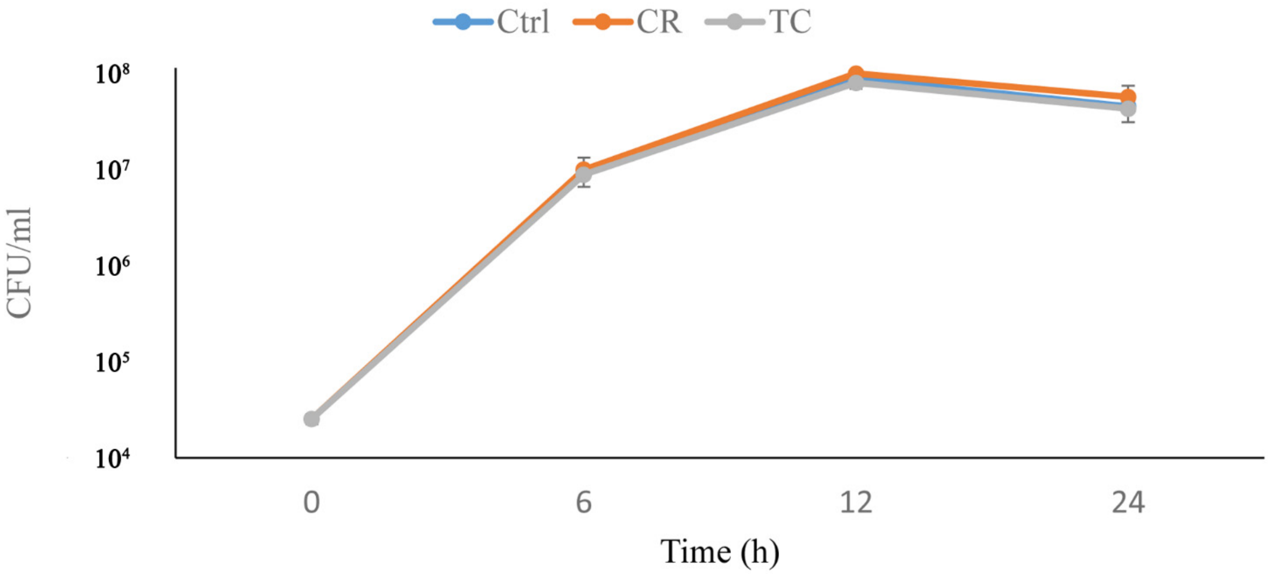

© 2014 by the authors; licensee MDPI, Basel, Switzerland. This article is an open access article distributed under the terms and conditions of the Creative Commons Attribution license (http://creativecommons.org/licenses/by/3.0/).
